# Efficacy of Probiotic Supplementation in the Management of Psoriasis: A Systematic Review

**DOI:** 10.7759/cureus.98265

**Published:** 2025-12-01

**Authors:** Muhammad Waqas, Inzamam Sarwar

**Affiliations:** 1 General Medicine, Cumberland Infirmary, Carlisle, GBR; 2 Respiratory Medicine, District Headquarters (DHQ) Allied Hospital 2, Faisalabad, PAK

**Keywords:** gastrointestinal microbiome, probiotics, psoriasis, quality of life, synbiotics

## Abstract

Psoriasis is a chronic immune-mediated skin disease that is increasingly associated with alterations in gut microbiota through the gut-skin axis. This systematic review assessed the efficacy of probiotic supplementation in managing psoriasis. A comprehensive search was performed across PubMed, Scopus, and the Cochrane Library for studies published between 2010 and 2025. After screening 688 unique records and removing duplicates and incomplete entries, 10 studies were found relevant, and eight with accessible full texts were included for qualitative synthesis. Across included randomized controlled and observational studies, probiotic or synbiotic supplementation either alone or as an adjunct to topical or systemic therapy was associated with reductions in Psoriasis Area and Severity Index (PASI) and Dermatology Life Quality Index (DLQI) scores. Several included studies also reported improvements in inflammatory biomarkers and gut microbiota composition, which were narratively presented in this review. Due to heterogeneity in study designs and interventions, findings were synthesized narratively. Multistrain formulations containing *Lactobacillus*, *Bifidobacterium*, or *Lactiplantibacillus plantarum* consistently demonstrated clinical benefits, whereas single-strain products yielded variable outcomes. All interventions were well tolerated, with only mild gastrointestinal discomfort noted in a small minority of participants. The available evidence suggests that probiotic supplementation may reduce disease severity, improve quality of life, and support immune and barrier function in individuals with psoriasis. The therapeutic effect appears strain-dependent and more pronounced in mild-to-moderate disease. Larger, well-controlled trials with standardized probiotic strains, defined dosages, and longer follow-up are needed to clarify the long-term clinical value of probiotics as adjunctive therapy in psoriasis management.

## Introduction and background

Psoriasis is a chronic, immune-mediated inflammatory skin disorder that affects nearly 43 million people worldwide. It presents with erythematous, scaly plaques that appear on different body regions and follow a relapsing-remitting pattern. The global prevalence ranges from 0.9% to 8.5% in adults and 0% to 2.1% in children. The disease imposes a significant physical, psychological, and socioeconomic burden, often disrupting quality of life and daily functioning [1,2]. Although psoriasis primarily affects the skin, it is increasingly recognized as a systemic condition associated with cardiometabolic disorders such as obesity, dyslipidemia, diabetes mellitus, and inflammatory bowel disease. These associations suggest shared immune mechanisms driven by chronic systemic inflammation [3,4].

The pathogenesis involves complex interactions among genetic predisposition, environmental triggers, and immune dysregulation. Overactivation of dendritic cells and T-helper lymphocytes (Th1 and Th17) stimulates the excessive release of cytokines, including tumor necrosis factor-α (TNF-α), interleukin-17 (IL-17), and interleukin-23 (IL-23). These cytokines promote keratinocyte proliferation and angiogenesis, sustaining a self-perpetuating inflammatory cycle in the skin. Studies suggest that probiotics may influence these inflammatory pathways by supporting the production of short-chain fatty acids (SCFAs), promoting regulatory T-cell activity, and enhancing intestinal barrier function. Standard therapies, including topical corticosteroids, vitamin D analogues, phototherapy, systemic immunosuppressants, and biologics targeting TNF-α, IL-17, or IL-23, help control disease activity. Yet, long-term use is often restricted by cost, potential toxicity, and declining efficacy, creating a need for safer, affordable adjunctive treatments [5-9].

Recent research has drawn attention to the gut-skin axis, a bidirectional communication pathway connecting intestinal microbiota with skin immune and inflammatory responses [10-12]. The gut microbiota regulates host immunity through metabolites, such as SCFAs, the modulation of regulatory T cells, and the maintenance of intestinal barrier integrity. Microbial imbalance, or dysbiosis, increases exposure to bacterial antigens, thereby enhancing Th17 activation and cytokine production associated with psoriasis [13,14]. Studies have reported reduced microbial diversity and altered *Firmicutes*/*Bacteroidetes* ratios in patients with psoriasis, suggesting that gut imbalance may contribute to disease severity [15].

Probiotics, live microorganisms that benefit the host when consumed in adequate amounts, have been proposed as potential modulators of systemic and cutaneous inflammation. Strains such as *Lactobacillus*, *Bifidobacterium*, and *Streptococcus thermophilus *help restore gut balance, suppress harmful bacteria, and regulate immune signaling. They reduce pro-inflammatory cytokines (IL-6, IL-17, TNF-α) and enhance anti-inflammatory mediators, such as IL-10 and transforming growth factor-β (TGF-β), thereby supporting immune tolerance. Probiotics also strengthen intestinal tight junctions, reducing permeability and systemic inflammation. Current clinical evidence focuses on immunocompetent individuals, and none of the included studies evaluated probiotic effects specifically in immunocompromised patients; therefore, their safety and immunologic impact in such populations remain uncertain [5-9].

Preclinical evidence supports these mechanisms. In animal models, oral *Lactobacillus *administration reduced psoriasiform inflammation by promoting Foxp3+ regulatory T-cell infiltration and limiting keratinocyte apoptosis [16]. Human studies show similar effects. Several randomized controlled trials (RCTs) demonstrated significant improvement in Psoriasis Area and Severity Index (PASI) and Dermatology Life Quality Index (DLQI) among participants receiving probiotics compared with placebo. These improvements were often associated with lower inflammatory marker levels and increased gut microbial diversity [17,18]. Zeng et al. reported that probiotics improved PASI scores in pooled RCTs, while Zhu et al. observed consistent though moderate benefits across various probiotic strains. In another meta-analysis, Wei et al. found that probiotic supplementation produced a standardized mean difference of −1.40 in PASI and −0.92 in DLQI compared with placebo, indicating clinically meaningful improvement without major adverse events. These results support probiotics as a safe adjunct to conventional psoriasis treatment [5,9,19].

Despite these promising results, certain challenges remain. Differences in probiotic species, dosage, duration, and concurrent therapies make comparisons across studies difficult. Most trials are small and short-term, limiting the assessment of sustained benefits and relapse prevention. Variations in diet, microbiome composition, and disease subtype may also influence therapeutic response. This systematic review was undertaken to evaluate the efficacy of probiotic supplementation in psoriasis management by synthesizing evidence from clinical studies, including RCTs, controlled trials, and prospective comparative studies that reported relevant clinical outcomes. The objective was to determine the outcomes reported in adults with psoriasis, receiving oral probiotics or synbiotics, compared with placebo or standard care, focusing on changes in PASI, DLQI, and inflammatory markers. Clarifying the role of probiotics within the gut-skin axis may guide the development of targeted, microbiome-based therapies for chronic inflammatory skin diseases.

## Review

Methodology

Search Strategy and Study Selection

This systematic review was conducted in 2025, following the Preferred Reporting Items for Systematic Reviews and Meta-Analyses (PRISMA) 2020 guidelines. An extensive search was performed across PubMed/MEDLINE, Scopus, and the Cochrane Library (CENTRAL), covering studies published between January 1, 2010, and December 31, 2025. The final search was carried out on October 27, 2025. This timeframe was selected to capture contemporary literature on the gut-skin axis, a concept that has gained increasing scientific attention since 2010. The search strategy combined Medical Subject Headings (MeSH) and free-text keywords related to psoriasis, probiotics, and randomized clinical trials, with Boolean operators (AND, OR) used to maximize retrieval and minimize omission. Equivalent terms such as "synbiotic", "Lactobacillus", "Bifidobacterium", and "gut microbiota" were also incorporated. Across all databases, a total of 875 records were retrieved before duplicate removal. Duplicates were identified through automated database tools and manual cross-checking and were removed prior to screening. Missing data were not pursued through author contact; records lacking essential bibliographic information were excluded.

**Figure 1:**
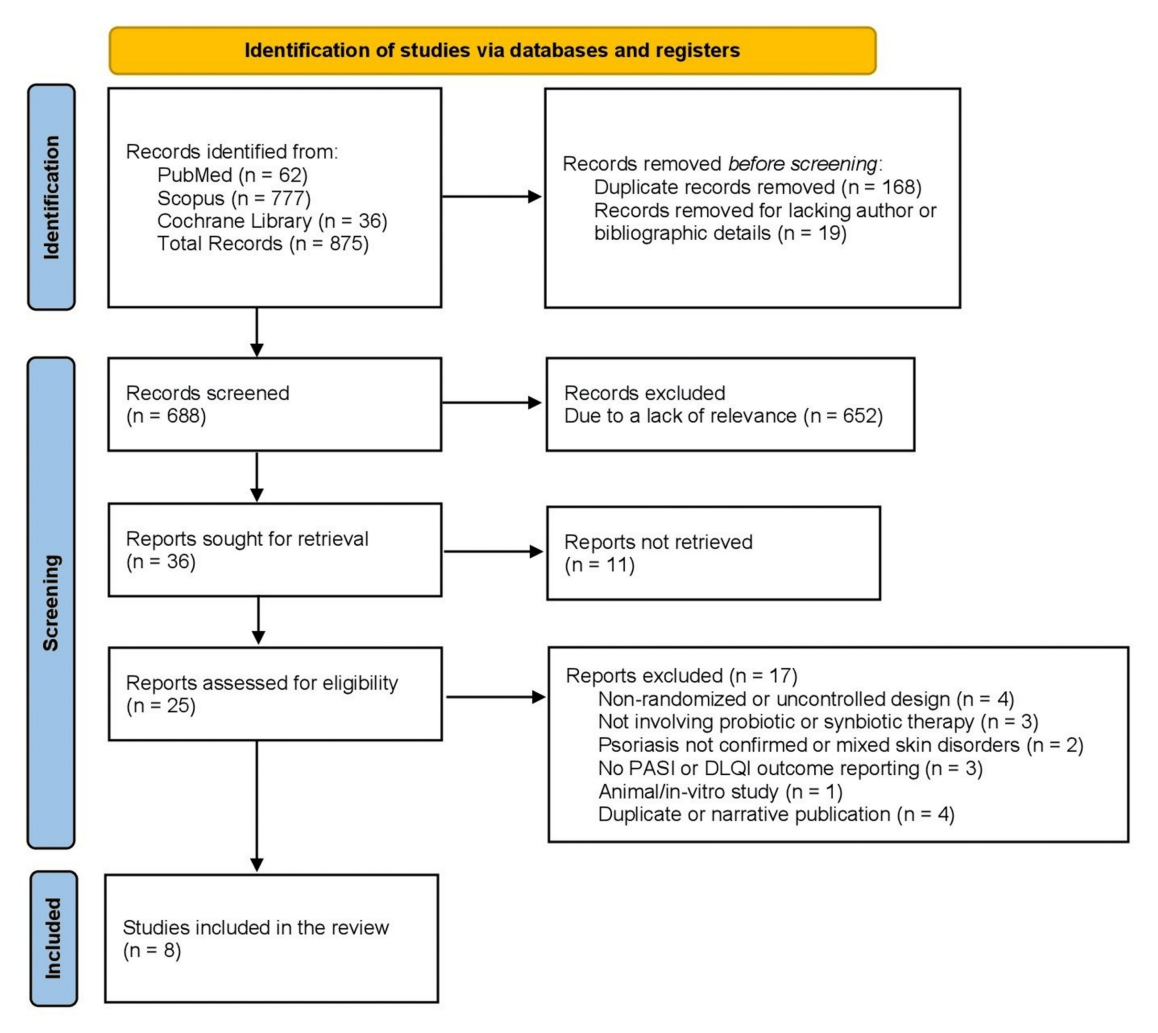
PRISMA flow diagram of the study selection process PRISMA: Preferred Reporting Items for Systematic Reviews and Meta-Analyses; PASI: Psoriasis Area and Severity Index; DLQI: Dermatology Life Quality Index

Two independent reviewers screened all retrieved articles by title and abstract based on predefined inclusion and exclusion criteria, and any discrepancies were resolved through discussion. Ten studies were identified as relevant, of which eight had full-text availability and were included in the final qualitative synthesis. The remaining two relevant studies could not be accessed in full text. The study selection process is illustrated in the PRISMA flow diagram, as shown in Figure 1, showing the number of records identified, screened, excluded, and included at each stage.

Inclusion and Exclusion Criteria

Studies were included if they met the following criteria: (1) human clinical trials enrolling adults diagnosed with any subtype of psoriasis (plaque, guttate, or pustular); (2) use of oral probiotic or synbiotic therapy as monotherapy or as an adjunct to standard dermatologic care; (3) comparison with placebo, no intervention, or standard therapy alone; and (4) reporting of clinical or patient-reported outcomes, particularly PASI, DLQI, or Visual Analogue Scale (VAS) scores. Both probiotic and synbiotic (probiotic + prebiotic) oral formulations were eligible for inclusion. Eligible study designs included RCTs, controlled clinical trials, and prospective open-label or longitudinal comparative studies where randomization was not feasible but clinical endpoints were reported.

Exclusion criteria encompassed animal or in vitro studies, reviews, editorials, and protocols without results. Studies focusing exclusively on microbiome characterization or biochemical parameters without clinical outcome data were also excluded. Trials that did not specify probiotic strain, dose, or treatment duration were omitted.

Data Extraction

All eligible articles were reviewed independently by two investigators using a standardized data extraction form. Extracted variables included author and publication year, country, study design, sample size, psoriasis subtype, intervention details (strain composition, dosage, and duration), comparator treatment, and concomitant therapy. Primary outcomes (PASI, DLQI, and VAS) and secondary endpoints (inflammatory biomarkers such as IL-6, IL-17, IL-10, high-sensitivity C-reactive protein (hs-CRP), malondialdehyde (MDA), lipopolysaccharide, and Foxp3) were systematically recorded along with adverse event profiles. A formal risk-of-bias tool (e.g., Cochrane RoB, Jadad, or Newcastle-Ottawa Scale) was not applied; instead, methodological quality was assessed narratively based on study design, participant selection, blinding, and reporting clarity.

Quality Assessment

Each included study was carefully reviewed for methodological soundness, focusing on study design, participant selection, randomization, and reporting clarity. Most trials were of moderate quality, with limitations mainly related to small sample sizes and lack of blinding in some studies. No study was excluded on the basis of quality.

Data Synthesis

Given the clinical and methodological heterogeneity across included trials involving differences in probiotic strains, dosages, duration of treatment (8-24 weeks), and adjunctive therapies, a meta-analysis was not performed. Instead, results were summarized narratively, focusing on the direction and magnitude of treatment effects across standardized outcomes (PASI, DLQI). Where available, improvements in inflammatory markers were noted to highlight biological plausibility.

Ethical Considerations and Registration

As this work involved secondary analysis of published literature, no ethical approval was required. The review protocol was not registered in the International Prospective Register of Systematic Reviews (PROSPERO); this decision was made early in the project due to limited time and resources, including the absence of dedicated funding for administrative research support. Nevertheless, methodological transparency was ensured through the detailed documentation of the search strategy, inclusion criteria, and PRISMA flow process.

Results

A total of 10 studies met the eligibility criteria, of which eight provided complete full texts suitable for detailed analysis. The included studies, presented in Table 1, were published between 2019 and 2024, collectively enrolling more than 700 participants with various forms of psoriasis. Most RCTs have demonstrated a significant reduction in PASI and DLQI scores among participants receiving probiotic or synbiotic supplementation compared with those receiving placebo or standard care, indicating meaningful clinical improvement.

**Table 1:** Summary of clinical studies evaluating the efficacy of probiotic or synbiotic supplementation in patients with psoriasis RCT: randomized controlled trial; PASI: Psoriasis Area and Severity Index; PGA: Physician Global Assessment; AE: adverse event; HC: hydrocortisone; VAS: Visual Analogue Scale; DLQI: Dermatology Life Quality Index; PsA: psoriatic arthritis; CFU: colony-forming unit; PSS: Psoriasis Symptom Scale; BDI: Beck Depression Inventory; hs-CRP: high-sensitivity C-reactive protein; IL: interleukin; MDA: malondialdehyde; TAC: total antioxidant capacity; QoL: quality of life; SOC: standard of care; BSA: body surface area; ns: not significant; ITT: intention-to-treat analysis; PP: per-protocol analysis

Study (year) and location	Intervention (strain)	Design and duration	Groups/N	Primary results	Safety	Conclusion	Notes
Navarro-López et al., 2019 (Spain) [20,21]	Multistrain probiotic (*Bifidobacterium longum*, *Lactobacillus rhamnosus*, *L. paracasei*)	RCT, double-blind, placebo-controlled, 12 weeks + 6-month follow-up	Probiotic (n=45) vs. placebo (n=43) (adults with plaque psoriasis)	PASI 75: 66.7% vs. 41.9% (p<0.05). PGA 0/1: 48.9% vs. 30.2%. Lower relapse over 6 months. Gut microbiota modulation confirmed	No major AEs reported	Probiotics improved clinical indices and reduced relapse risk	Co-treatment was topical steroids per protocol description; full strain list and dose in paper
Akbarzadeh et al., 2022 (Iran) [22]	Lactocare® synbiotic + topical hydrocortisone	RCT, double-blind, placebo-controlled, 12 weeks	Probiotic + HC (n=25) vs. placebo + HC (n=27)	Between-group: PASI 4.08±0.28 vs. 6.19±0.39 at week 12 (p<0.001); VAS 32.24±4.29 vs. 48.11±4.88 (p=0.019); DLQI 6.56±0.63 vs. 8.92±0.71 (p=0.017). Intragroup: progressive PASI/VAS/DLQI reductions from baseline → weeks 4/8/12	Well tolerated; no conflicts declared	Adjunct synbiotic significantly improved PASI, DLQI, and VAS at 12 weeks	Effect emerged by week 12 (weeks 4/8 not significant between groups)
Buhaș et al., 2023 (Romania) [23]	Spore-based *Bacillus *probiotic mix + prebiotics	Open-label, controlled (non-randomized), 12 weeks	Intervention (n=42) vs. control (n=21)	Intervention showed better PASI, DLQI, inflammatory markers, and skin thickness vs. control; in a subset (15/42), microbiota shifted to an anti-inflammatory profile after 12 weeks	No AEs reported	Signals benefit; baseline imbalances limit certainty	Groups differed at baseline (age, severity, nail disease, PsA)
Moludi et al., 2021 (Iran) [24]	Multistrain *Lactobacillus*/*Bifidobacterium* capsules (~1.8×10^9^ CFU; twice a day)	RCT, double-blind, placebo-controlled, 8 weeks	Probiotic (n=25) vs. placebo (n=25)	PASI Δ −5.26±3.75 vs. −0.48±1.37 (p=0.049); PSS Δ −4.85±3.10 vs. −0.43±0.80 (p=0.047); DLQI Δ −9.50±4.1 vs. 0.12±0.6 (p=0.045); BDI improved (p=0.017); biomarkers: ↓hs-CRP, ↓IL-6, ↓MDA, ↑TAC (all p≤0.05)	No serious AEs	Short-term probiotics improved symptoms, QoL, and inflammatory/oxidative markers	Single center; young cohort
Gilli et al., 2023 (Brazil) [25]	Lactobacillus rhamnosus	RCT, placebo-controlled, 60 days	Probiotic (n=18) vs. placebo (n=17)	Significant ↓PASI, ↓BSA, ↓DLQI in probiotic group; IL-17/IL-23 unchanged	Minimal AEs; well tolerated	Clinical improvement over 60 days without serum IL-17/IL-23 change	Mixed common + palmoplantar psoriasis
Suriano et al., 2023 (Brazil) [17]	*Lactobacillus rhamnosus *as adjuvant to SOC	RCT, parallel, double-blind, 6 months	Probiotic (n=50) vs. placebo (n=53)	Within-group: experimental PASI Δ −1.58 (p=0.105), DLQI Δ +0.05 (p=0.873), not significant; controls had small but significant improvements. Between group: ns (p=0.620)	AEs not detailed; no major issues stated	No added clinical benefit over SOC at 6 months	Tertiary referral setting; ITT and PP presented
Umborowati et al., 2024 (Indonesia) [16]	*Lactiplantibacillus plantarum *IS-10506 (2×10^10^ CFU/day) + standard topical therapy	RCT, double-blind, placebo-controlled, 12 weeks (+6-month follow-up)	Probiotic (n=24) vs. placebo (n=25)	PASI lower in probiotic at week 6 (p=0.024) and week 12 (p=0.049); week 12 DLQI numerically lower (7.57±5.77 vs. 7.79±5.48). IL-17 ↓ (p=0.013); IL-10 ↑ (p≤0.001); Foxp3 ↑ (p=0.048). Flares at 6 months: 52.2% vs. 79.2%	Mild bowel habit change: 2 probiotics, 1 placebo; mild nausea: 1 placebo	Strain IS-10506 improved clinical and immune markers; fewer flares	Both arms used a topical corticosteroid + emollient
Zangrilli et al., 2022 (Italy) [18]	*Streptococcus salivarius *K-12	Longitudinal case-control (treated vs. untreated), 24 months (outcomes at 24 weeks and beyond)	Treated (n=100) vs. control (n=98)	At week 24 in treated: PASI 75 84%, PASI 90 75%, PASI 100 55% vs. controls 42.8%, 30.6%, 21.4% (χ²; p=0.01). Sustained effects during follow-up; well tolerated	No AEs observed	Large signal of benefit, but non-randomized design limits inference	Baseline PASI higher in treated; tonsil-immunity hypothesis explored

Discussion

This systematic review evaluated the efficacy of probiotic supplementation as an adjunct or independent therapy in psoriasis. Evidence suggests that probiotics may improve clinical severity, quality of life, and inflammatory biomarkers, though the degree of benefit varies with strain type, study design, and treatment duration. Psoriasis is a chronic, immune-mediated skin disease with multifactorial pathogenesis involving genetic predisposition, dysregulated immune signaling, and microbial imbalance. The gut-skin axis provides a biological rationale for probiotic therapy, as intestinal dysbiosis may trigger systemic inflammation through the disruption of epithelial integrity and immune modulation. Experimental work has demonstrated altered microbial composition in psoriasis, including reduced *Firmicutes *and increased *Bacteroidetes *and *Actinobacteria*, which correlate with disease activity [26,27]. The restoration of microbial diversity using probiotics may attenuate systemic inflammation by modulating interleukin and tumor necrosis factor pathways, enhancing barrier function, and promoting regulatory T-cell differentiation [28,29].

Among RCTs, the Spanish study by Navarro-López et al. provided early evidence that multistrain probiotics containing *Bifidobacterium longum*, *Lactobacillus rhamnosus*, and *L. paracasei* can significantly improve psoriasis outcomes. After 12 weeks, 66.7% of probiotic-treated adults achieved PASI 75 compared with 41.9% in the placebo group, with a lower relapse rate at six months. These findings supported the therapeutic potential of probiotic combinations targeting both gut and skin inflammation. Similarly, the Iranian trial by Akbarzadeh et al. using a synbiotic capsule (Lactocare®) with topical hydrocortisone demonstrated meaningful reductions in PASI (4.08±0.28 vs. 6.19±0.39), VAS, and DLQI scores at week 12 compared with placebo. These results confirm that probiotics can enhance the efficacy of standard topical therapy through immunomodulatory and anti-oxidative mechanisms [20-22].

Moludi et al. reported that a multistrain *Lactobacillus*/*Bifidobacterium* probiotic drink administered for eight weeks produced significant decreases in PASI and DLQI, together with improvements in oxidative and inflammatory biomarkers such as MDA, hs-CRP, IL-6, and total antioxidant capacity. These biological effects align with experimental data showing that probiotics suppress nuclear factor-κB signaling and downregulate pro-inflammatory cytokines implicated in keratinocyte proliferation. A later Iranian study by the same group observed similar reductions in PASI (mean Δ -5.06±2.10) and serum lipopolysaccharide levels, suggesting improved intestinal barrier integrity [24,30].

In contrast, Suriano et al. conducted a well-designed, double-blind RCT in Brazil assessing *Lactobacillus rhamnosus *as an adjunct to standard of care in plaque psoriasis. Although within-group improvement occurred, the between-group difference was not significant. The investigators proposed that concomitant use of systemic or topical agents may have masked any additive probiotic effect. Similarly, Gilli et al. found clinical improvement with *L. rhamnosus *supplementation over 60 days but without parallel changes in IL-17 and IL-23 levels, suggesting a limited immunologic impact. These findings indicate that probiotic strain selection, dose, and duration critically determine outcomes [17,25].

More encouraging data emerged from Asia. Umborowati et al. studied *Lactiplantibacillus plantarum *IS-10506, an Indonesian-origin strain, in 49 patients with mild-to-moderate psoriasis vulgaris. Significant reductions in PASI at week 6 and week 12 were accompanied by lower IL-17 and higher IL-10 and Foxp3 levels, reflecting immune rebalancing. At the six-month follow-up, relapse probability was lower in the probiotic group (52.2% vs. 79.2%), reinforcing the role of long-term microbial modulation in disease control. Zangrilli et al. reported a large case-control cohort using *Streptococcus salivarius *K-12, in which 83.7% achieved PASI 100 at 24 weeks with no adverse events. Though non-randomized, this study highlights potential benefits from probiotics targeting oropharyngeal dysbiosis and post-streptococcal immunity [16,18].

The beneficial effects observed across studies can be explained by several biological pathways. Probiotics modulate innate and adaptive immunity through toll-like receptor signaling, leading to the downregulation of pro-inflammatory cytokines, such as TNF-α, IL-6, and IL-23, and the upregulation of anti-inflammatory mediators, including IL-10 and TGF-β [26]. They also enhance epithelial barrier integrity and reduce systemic endotoxemia, as evidenced by decreased circulating lipopolysaccharide levels in probiotic-treated psoriasis cohorts [24]. The increase in total antioxidant capacity and reduction in MDA observed in these trials further suggest the restoration of oxidative balance. Recent neuroimmunologic findings indicate that the gut microbiome may also influence stress-related pathways and skin inflammation through the microbiota-brain axis [31]. This provides an additional rationale for probiotics improving psychological comorbidities such as anxiety and depression, frequently associated with psoriasis, reflected by improved Beck Depression Inventory (BDI) scores in Moludi et al.'s trial [24].

Although most trials demonstrated improvement in PASI and DLQI, the magnitude of benefit varied. Discrepancies are likely due to heterogeneity in probiotic composition, dosage, and baseline disease severity. Multistrain formulations appeared more effective than single-strain products, possibly due to the synergistic modulation of gut flora. Duration longer than 12 weeks tended to produce sustained benefits, supporting the concept that probiotics require time to re-establish microbial equilibrium. Variability in concomitant treatments also influenced outcomes. Trials combining probiotics with topical corticosteroids or emollients showed additive effects, whereas those conducted on patients receiving systemic immunosuppressants did not demonstrate additional improvement [16,17,22]. The lack of standardized outcome measures beyond PASI and DLQI further complicates direct comparison.

The gut-skin immune crosstalk involves several mechanisms. Dysbiosis can promote Th17-driven inflammation and keratinocyte hyperproliferation. Probiotics restore microbial balance, increase the production of SCFAs such as butyrate, and modulate dendritic cell function, leading to the expansion of regulatory T cells [27]. This immunologic shift aligns with Umborowati et al.'s finding of increased Foxp3 expression and reduced IL-17 in treated patients. Moreover, probiotic supplementation may normalize toll-like receptor signaling, preventing the excessive activation of nuclear factor-κB and subsequent cytokine release [26]. The observed reductions in hs-CRP and IL-6 further support systemic anti-inflammatory activity. Beyond immune modulation, probiotics may improve gut barrier permeability, reducing the translocation of bacterial toxins and endotoxins implicated in systemic flare-ups. Different probiotic strains appear to exert distinct effects. *Lactobacillus rhamnosus *and *Bifidobacterium longum *primarily modulate mucosal immunity and improve epithelial integrity. *L. plantarum *shows stronger anti-inflammatory activity via IL-10 induction, while *S. salivarius *K-12 may influence mucosal immunity through the inhibition of *Streptococcus pyogenes *superantigens, a proposed trigger in guttate psoriasis. The consistent safety across all studies reinforces the tolerability of probiotics, with only mild gastrointestinal symptoms reported. Findings from other immune-mediated inflammatory diseases, including inflammatory bowel disease and rheumatoid arthritis, support probiotic efficacy in reducing cytokine levels and improving mucosal health [29]. This cross-disease consistency strengthens the biological plausibility of probiotics as adjunct therapy for psoriasis. Animal models further demonstrate that probiotic-induced modulation of the gut microbiota alleviates imiquimod-induced psoriatic dermatitis and restores IL-10/IL-17 balance [28]. Such experimental evidence complements the human clinical data.

Limitations

Most of the included studies were conducted at single centers with relatively short follow-up periods, limiting the strength of evidence. Study protocols varied in design, probiotic formulation, dosage, and treatment duration, making comparisons less consistent. The diversity of bacterial strains and incomplete reporting of colony-forming units reduced reproducibility across trials. Dietary intake and concurrent treatments, which may influence gut microbiota, were seldom assessed. Standardized outcome measures beyond PASI and DLQI were infrequently applied, and biomarker assessment was limited. Publication bias cannot be ruled out, as positive findings are more likely to be published; however, a formal assessment of meta-bias was not feasible because no meta-analysis could be performed. The predominance of data from a few geographic regions also restricts the generalizability of findings.

## Conclusions

Probiotic supplementation may improve psoriasis severity, quality of life, and inflammatory markers, with a generally favorable safety profile. Across available evidence, the most consistent signals were observed with multistrain formulations and with *Lactiplantibacillus plantarum *IS-10506, particularly in studies with treatment durations of 12 weeks or longer. Probiotics may function as supportive therapy alongside standard management for mild-to-moderate psoriasis, although variability across strains and study designs limits definitive conclusions. Larger, multicenter trials with standardized strains, clearly defined dosages, and extended follow-up are needed to confirm long-term benefits and identify patients most likely to respond. While emerging data on the gut-skin axis are promising, probiotics should still be considered an adjunct rather than a standalone therapy.
